# Avoid Postoperative Pain To Prevent Its Chronification: A Narrative Review

**DOI:** 10.7759/cureus.22243

**Published:** 2022-02-15

**Authors:** Antonio Montero Matamala, Magdi Hanna, Serge Perrot, Giustino Varrassi

**Affiliations:** 1 Department of Surgery, University of Lleida, Lleida, ESP; 2 Department of Anesthesiology, University of London, London, GBR; 3 Rheumatology, Cochin University Hospital, Paris, FRA; 4 Research, Paolo Procacci Foundation, Roma, ITA

**Keywords:** dexketoprofen, tramadol, drugs fixed dose combination (fdc), multimodal analgesia, pain chronification, acute pain

## Abstract

Acute postoperative pain is a normal and expected part of the patient’s postsurgical trajectory, and its intensity, severity, and duration vary with surgery-related and patient factors. In a subset of patients, postoperative pain does not resolve as the tissue heals but instead transitions to chronic postoperative pain, a challenging condition to treat and one associated with decreased quality of life, sleep and mood disorders, and neuropathy. Promptly and adequately treating acute postoperative pain can reduce the risk that it will transition into chronic postoperative pain. Numerous agents are available that may help treat postoperative pain, including nonsteroidal anti-inflammatory drugs, opioids, antidepressants, anticonvulsants, and others. In this connection, it is also important to consider patient factors, such as mental health status and comorbidities, as well as the type and duration of surgery. A multimodal approach is recommended, which uses two or more agents with complementary mechanisms of action, working at different targets. Multimodal analgesia may also reduce adverse events and lessen opioid consumption after surgery. A particularly useful fixed-dose combination product is dexketoprofen/tramadol (DEX-TRA), which is safe and effective in numerous clinical trials. This review is based on a presentation from the Roma Pain Days scientific sessions of 2021.

## Introduction and background

As part of the Roma Pain Days session on Multimodal Analgesia for the Effective Treatment of Acute Pain, a presentation was given on acute postoperative pain; and how prompt, effective treatment may reduce the risk that persistent post-surgical pain will develop. Post-surgical pain typically follows a predictable and surgery-specific trajectory, with pain most intense immediately after surgery and lessening gradually as the tissue heals. Acute post-surgical pain is normal and expected, but it can transition into more persistent or even chronic post-surgical pain [1]. Risk factors for chronic post-surgical pain include type and duration of surgery, genetics, anesthesia, and analgesia used, patient psychology and mental health status, surgical complications, underlying disease, and comorbidities [2]. The current strategy to prevent persistent postoperative pain is to promptly and effectively manage acute postoperative pain [3].

The presentation aimed to better elucidate the potential transition of acute postoperative pain into chronic pain syndromes. Postoperative pain is prevalent [1], and better understanding is needed to avoid its chronification. This review describes a large and heterogenous patient population, namely those who undergo surgery.

## Review

Acute post-surgical pain is normal and expected in approximately the first month following surgery. The duration and intensity of post-surgical pain vary, depending on the type of surgery and its duration, patient factors, and whether or not there were surgical complications [4]. If pain persists after one month, it may be considered persistent or subacute post-surgical pain. This condition is not rare, as it occurs in about 10% to 50% of surgical patients. Persistent postsurgical pain has been associated with increased morbidity, decreased patient satisfaction, and an economic burden on the patient and the healthcare system [5]. In 2% to 14% of surgical patients, pain following surgery persists for ≥ three months and is known as chronic postoperative pain. Chronic postoperative pain is associated with decreased quality of life, sleep disorders, mood disorders, and neuropathic symptoms [6].

Risk factors have been identified for the development of chronic postoperative pain and include age, genetic factors, the presence of pre-existing pain, severe acute postoperative pain in the first 24 hours after surgery, surgery-related factors, and psychological factors [4,7-11]. With respect to age, younger patients are at higher risk for chronic postoperative pain than older individuals in many types of surgery. [9] In fact, the risk of chronic postoperative pain after mastectomy among those between the ages of 30 and 49 years is more than double that of those ≥ 70 years [9]. Among the surgery-related factors is the type of surgery, duration of surgery, location and type of incision, and the surgeon’s experience [11]. Psychological factors that put a patient at risk for chronic postoperative pain are pre-operative anxiety and catastrophizing, that is, entertaining and focusing on exaggerated negative ideas about the surgery [10]. Pre-existing pain must also be considered a risk factor. This may include intense pain immediately before surgery or a chronic pain condition that lasted more than six months in the surgical patient [12].

While not all risk factors are modifiable, there do exist opportunities for clinical interventions. For example, patients suffering severe pain immediately before surgery should be treated to control their pain levels [7]. Chronic pain patients should also be given adequate analgesia so that they do not undergo surgery with pre-existing pain [7]. Acute pain in the first 24 hours after surgery must be controlled immediately, as severe pain in this window of time has been associated with chronic postoperative pain [7]. Psychological factors may not always be modifiable but should be recognized and addressed to the greatest extent possible by patient education, counseling, encouragement by the clinical team, or engaging the family or friends of the patient to provide strong, positive support.

The surgical patient follows a relatively predictable pathway from pre-operative encounter to discharge, and each of the steps along the way allows for opportunities to defend against chronic postoperative pain [13]. 

Current pharmacologic strategies

Numerous drugs and techniques have been assessed for control of perioperative and postoperative pain, including, but not limited to, opioids, nonsteroidal anti-inflammatory drugs (NSAIDs), antidepressants, anticonvulsants, such as gabapentin or pregabalin, clonidine, intravenous (IV) ketamine, local anesthesia, and other agents. Multimodal analgesia refers to the use of more than one agent with complementary mechanisms of action [14,15]. There are few studies of these agents in post-surgical pain because such studies would necessarily have to be of very long duration (≥ six months), and results can vary due to many factors, including the patient’s anxiety levels and degree of fearfulness. While more studies are needed, it is not likely that even with many new studies, there would be unequivocal guidance for the range of patients in all types of surgeries.

Venlafaxine, a selective serotonin and norepinephrine reuptake inhibitor (SSNRI), has been compared to gabapentin and placebo in a randomized controlled trial of 150 patients undergoing partial or radical mastectomy with axillary dissection [16]. The patients were randomized into three arms: venlafaxine extended-release 37.5 mg/day; gabapentin 300 mg/day; or placebo for 10 days starting the night before the surgery. The pain was assessed at rest and during movement at four, 12, and 24 hours post-operatively, then daily to the tenth postoperative day, and again at six months. Both agents reduced pain in the immediate postoperative period in a similar fashion but with different patterns. Gabapentin reduced pain during movement from day two to day 10; venlafaxine reduced pain during movement on days eight, nine, and 10. Both venlafaxine and gabapentin reduced pain at rest similarly and reduced analgesic consumption compared to placebo. At six months, the venlafaxine patients had significantly lower rates of chronic pain compared to gabapentin and placebo patients [16]. In this study, gabapentin conferred no benefits in the prevention of chronic postoperative pain.

As a class of drugs, antidepressants may not help treat postoperative pain. A review of antidepressants in surgical patients (15 studies on early postoperative pain, n=985; three studies on chronic postoperative pain, n=565) found that, as a class, there was insufficient evidence to recommend them for acute or chronic postoperative pain [17]. However, this review did find that venlafaxine alone conferred benefits concerning chronic postoperative pain.

Anticonvulsants are associated with equivocal evidence. A systematic review of 18 randomized clinical studies using pregabalin for surgical patients to prevent chronic post-surgical pain (n=1884) found moderate-quality evidence to suggest that pregabalin conferred no preventive effect [18]. A systematic review of using gabapentin to prevent chronification of postoperative pain examined five studies that assessed pain levels three months post-operatively and found that chronic pain rates did not differ between gabapentin and placebo patients [19]. However, this analysis found a single study in which administration of 1.2 g oral gabapentinoid before surgery and 300 mg/day for the first 30 days after surgery resulted in lower morphine consumption than placebo. Overall, gabapentin was not superior to a placebo at preventing chronic postoperative pain [19].

The α2-adrenoreceptors play a role in nerve lesions and the local infiltration of macrophages and lymphocytes, inhibiting the release of pro-inflammatory cytokines [20]. Clonidine and dexmedetomidine, α2-adrenergic agonists, have been studied with respect to neuropathic pain. In a study of colon surgery patients, subarachnoid clonidine 300 mg and bupivacaine 10 mg versus bupivacaine 10 mg alone found that the use of clonidine plus bupivacaine was associated with a significantly reduced rate of chronic post-surgical pain at six and 12 months versus bupivacaine alone [21]. Further study is needed.

In a randomized, double-blind clinical trial of 36 patients undergoing breast surgery, patients received either an IV bolus of lidocaine 1.5 mg/kg followed by continuous infusion of lidocaine 1.5 mg/kg/h or similar amounts of saline. An infusion was initiated before general anesthesia was induced and stopped one hour after skin closure. Pain scores and consumption of analgesics were assessed at two, four, and 24 hours after surgery, then daily for one week, and finally at three months. Persistent post-surgical pain was reported at three months in 11.8% of the lidocaine group and 47.4% of the control group. None of the lidocaine patients reported any pain on movement at three months compared to 42% of the control group. Controls had higher rates of secondary hyperalgesia (area of hyperalgesia around the incision), but both groups had similar analgesic consumption [22]. This study did not follow patients beyond three months, so caution is appropriate in considering results.

A systematic review and meta-analysis compared the use of local anesthetics and regional anesthesia to conventional analgesia for surgical patients, assessing the development of persistent surgical pain at six and 12 months after surgery. The review utilized 23 randomized clinical trials with data for six months (n=1090) or 12 months (n=441) [23]. Perioperative epidural anesthesia, when used intraoperatively and post-operatively, reduces persistent pain following surgery. In breast surgery patients, paravertebral block prevents chronic postoperative pain in 20% of patients. The use of a continuous peripheral nerve block seems beneficial in reducing the development of postoperative pain. In iliac crest biopsy and breast surgery, the use of wound infiltration with local anesthetics reduces the rate of postoperative pain [23]. There is good evidence in support of the use of regional techniques to reduce acute postoperative pain, but their ability to decrease chronic post-surgical pain requires further study.

Ketamine shows promise for reducing chronic post-surgical pain, although studies to date have been few and may overstate treatment effects [24]. In particular, IV ketamine seems to significantly reduce the risk of chronic post-surgical pain at three or six months. The recommended dose is in the range of 0.25 to 0.75 mg/kg IV bolus followed by a continuous infusion of 2 to 7 µg/kg/min [24].

Other drugs have been studied for the prevention of persistent post-surgical pain: memantine, dextromethorphan, dexmedetomidine, mexiletine, nitrous oxide, and tumor necrosis factor (TNF)-α blockers. The results from these studies have been mixed, with no strong evidence in support of any of these drugs [25]. However, TNF-α blockers may be of particular interest as they inhibit microglial activation, which may play a role in chronic pain syndromes.

The multimodal analgesic approach for surgical patients utilizes two or more treatments for pain with complementary mechanisms of action, based on the idea that surgical pain is multimechanistic and these particular mechanisms must be individually addressed. NSAIDs are effective and widely used to reduce acute postoperative pain, but they do not reduce chronic post-surgical pain [26,27]. Opioid analgesics are effective postoperative pain relievers, but their use before surgery may increase the risk for chronic post-surgical pain [28]. Gynecological surgical patients taking opioids before surgery were twice as likely to report chronic post-surgical pain than those who had not taken opioids before surgery [29]. In addition, opioids are associated with numerous side effects, such as dizziness, nausea, vomiting, and constipation, which may be treatment-limiting [30].

**Figure 1:**
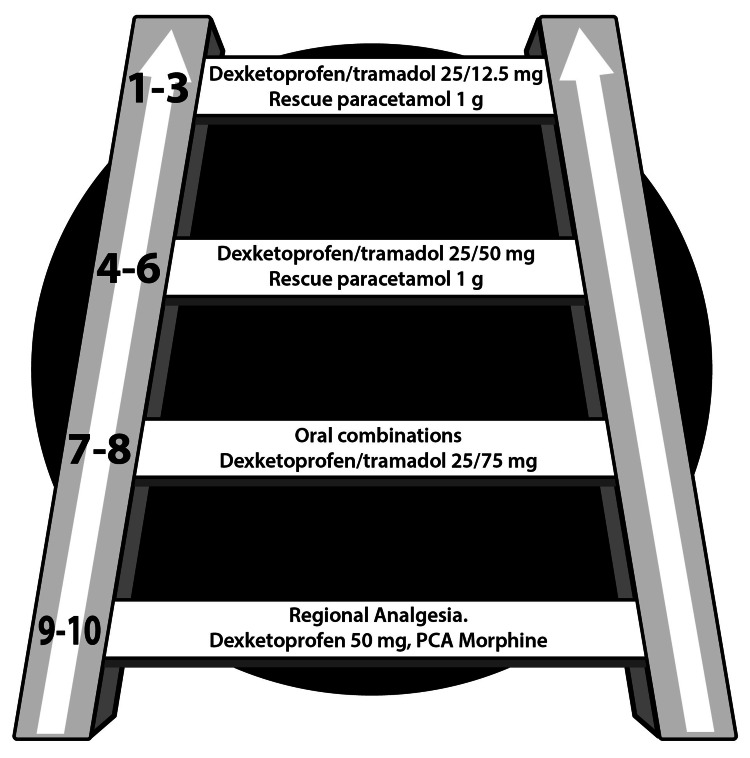
Postoperative analgesic ladder Analgesic regimen for surgical patients with pain intensity level shown on the left-hand side of each step. For the most severe pain, parenteral or regional analgesia is recommended. As the pain lessens, the patient can transition to fixed-dose combination oral products, in this case, dexketoprofen and tramadol, incrementally decreasing the dose as post-surgical pain decreases. When pain descends to level six or below, a lower dose oral product may be combined with rescue paracetamol (acetaminophen) as needed. Co-analgesics can be used in all steps of the ladder.

Effective multimodal analgesia regimens offer the benefits of fewer side effects and reduced opioid consumption without sacrificing analgesic efficacy [14,31-33]. It is not clear if multimodal post-operative analgesia can reduce chronic postoperative pain, even if it were assumed to be helpful in this regard; the exact peri-operative and post-operative analgesia regimens and their mechanisms of action are not elucidated [34]. For surgical patients, a “pain ladder” may illustrate how pain can be addressed based on its intensity, which typically decreases as the tissue heals (Figure 1). For surgeries that confer a particularly high risk for chronic post-surgical pain, ketamine and/or lidocaine may be used as well with a bolus dose followed by continuous infusion [26].

The analgesic protocol may further be adjusted based on the type of surgery and anticipated pain intensity levels. For example, knee arthroplasty is associated with high rates of postoperative pain, benefits from early mobilization and rehabilitation, and thus benefits from prompt, effective pain control. When considering pain control protocols, it is important to incorporate nonpharmacologic approaches as appropriate. An analgesic protocol appears in Table 1.

**Table 1:** Analgesic protocol for knee arthroplasty which incorporates both pharmacologic and nonpharmacologic steps.

Phase	Pharmacologic	Nonpharmacologic
Pre-operative	Two hours before surgery: Midazolam 7.5 mg Dexamethasone 0.1 mg/kg	Patient education counseling patient and family
Regional anesthesia	Spinal anesthesia Hyperbaric Bupivacaine 15 mg plus Sufentanil 2.5 µg	--
Intra-operative	Dexketorpofen 50 mg Lidocaine 1.5 mg/kg/h Ketamine 0.2 mg/kg Wound infilitration (local infiltration analgesia)	--
Post-operative	Adductor canal block: Dexketoprofen 50 mg and Tramadol 50 mg Oral fixed-dose combination: Dexketoprofen/Tramadol (DEX-TRA) 25/75 mg	Early ambulation Early rehabilitation
Post-discharge	Oral: DEX-TRA 25/75 mg every 12 h for 3 days Thereafter: Dexketoprofen 25 mg every 24 h for 5 days	Rehabilitation

The role of the combination of dexketoprofen/tramadol

Dexketoprofen trometamol is a modified nonselective inhibitor of both the cyclo-oxygenase (COX)-1 and COX-2 enzymes. It has demonstrated effectiveness in the treatment of acute pain and exerts an opioid-sparing effect when incorporated into multimodal regimens [35]. Its onset of action is rapid, < 30 minutes, and it offers a low reduction of the renal excretory rate [36]. Tramadol has dual mechanisms of action and can be effective in neuropathic pain conditions. The combination of dexketoprofen and tramadol allows for a smooth transition from parenteral administration to oral dosing; fixed-dose combination products are available in certain dose combinations to reduce the pill burden and improve patient acceptance [14]. The combination of dexketoprofen and tramadol is well tolerated [37]. (Table 2)

**Table 2:** Mutlimodal analgesia using a combination of dexketoprofen trometamol and tramadol.

	Dexketoprofen trometamol	Tramadol
Mechanism of action	Inhibits COX-1 and COX-2 Inhbitis prostaglandin (PG) E2 synthesis	µ opioid receptor agonist Inhibits serotonin and noradrenaline reuptake
Tmax	30 min (range 15-60)	Range 96-120 min
Bioavailability	High	70% to 90%
Metabolism	Hepatic Glucuronoconjugation Cytochrome (CY) P-2C8 and CYP-2C9	Hepatic Metabolized by CYP-2D6, N and O demethylation, active metabolite M1 O-dimethyltramadol
Excretion	Renal	Renal
Analgesic characteristics	Fast-acting central and peripheral anti-inflammatory effects	Long-lasting central

Many fixed-dose combination products offering multi-modal analgesia have established that they provide safe and effective postoperative analgesia [27]. (Table 3)

**Table 3:** Fixed-dose combination products for the treatment of moderate to severe acute postoperative pain.

	Combination	Doses
Moderate pain	Paracetamol/Codeine	500 mg/30 mg
	Paracetamol/Tramadol	325-600 mg/37.5-75 mg
	Ibuprofen/Codeine	400 mg/30 mg
	Ibuprofen/Oxycodone	400 mg/5 mg
Severe pain	Diclofenac/Tramadol	25 mg/25 mg
	Ketorolac/Tramadol	30 mg/75 mg
	Dexketoprofen/Tamadol	25/75 mg
	Celecoxib/Tramadol	200/75 mg

In a randomized, double-blind, double-dummy, parallel-group, placebo-controlled, single-dose study of 606 patients with moderate to severe dental pain after third molar extraction, patients were administered one of the following treatments for pain: monotherapies of dexketoprofen at doses of 12.5 or 25 mg; tramadol 37.5 or 75 mg; ibuprofen 400 mg as the active comparator; and placebo [38]. There were all four combination groups with dexketoprofen/tramadol (DEX-TRA) doses of 12.5/37.5 mg, 12.5/75 mg, 25 mg/37.5 mg, and 25 mg/75 mg, respectively. The primary endpoint of this single-dose study was > 50% pain reduction over six hours after oral surgery. The study arm with the highest proportion of responders, defined as those who met the primary endpoint, was 72% in the DEX-TRA 25/75 mg group with a number needed to treat (NNT) of 1.6. All four study arms using the combination product of DEX-TRA resulted in NNT values < 4. However, the NNT for tramadol 75 mg as monotherapy > six. No unusual adverse events were reported. All of the DEX-TRA combinations offered pain relief for a median of 8.1 hours [38].

The DEX-TRA fixed-dose combination product was evaluated in several clinical trials [38-44]. Efficacy and safety were confirmed for pain control following oral surgery [38,39], abdominal hysterectomy [40], and total hip arthroplasty [41]. In a posthoc analysis of two phase III clinical trials (DEX-TRA-04 and DEX-TRA-05), 933 patients were actively treated with DEX-TRA 25/75 mg, dexketoprofen monotherapy 25 mg, or tramadol monotherapy 100 mg and were evaluated for pain control over 56 hours. The fixed-dose combination product DEX-TRA 25/75 mg provided significantly superior analgesia over the other two products at 56 hours [42]. A systematic review and meta-analysis of three studies (n=1853) for acute postoperative pain after various surgeries found fixed-dose combination DEX-TRA 25/75 mg was effective, provided sustained pain control over eight hours or longer, and reduced analgesic consumption in the immediate postoperative period. About 10% of patients taking this product had side effects, mostly mild to moderate, with the most frequently reported being nausea, vomiting, and dizziness [43]. An expert consensus that was based on input from over 100 international pain experts found fixed-dose dexketoprofen and tramadol to be a safe, effective, well-tolerated, and long-lasting analgesic for moderate to severe acute pain [44].

Studies of chronic postoperative pain can be challenging to interpret because they may involve different types of surgery, various patient populations, and different follow-up periods. More study is warranted for chronic post-surgical pain and ways to synthesize the available and new data to draw meaningful clinical conclusions that are useful in real-world practice.

This article has certain limitations. It is based to a large extent on a presentation at a scientific session. It is a narrative review. 

## Conclusions

Chronic post-surgical pain is prevalent and possibly preventable. Surgeons can help reduce the risk of persistent post-surgical pain by minimizing surgical trauma. Anesthesiologists must be aware and proactive in working against chronification of post-surgical pain. Multimodal analgesia regimens are important, particularly when they can spare opioid consumption. A transition from IV to oral analgesia should be made as soon as it is safe and possible to do so. There is no optimal analgesic agent or pain control regimen that can reliably prevent acute pain from transitioning to chronic pain, but multiple studies corroborate the superior efficacy of pain control using two-drug combinations that have complementary mechanisms of action, such as DEX-TRA. Patient and clinician education is also important to raise awareness about persistent postoperative pain and steps to prevent it. 

## References

[1] Kehlet H, Jensen TS, Woolf CJ (2006). Persistent postsurgical pain: risk factors and prevention. Lancet.

[2] Fregoso G, Wang A, Tseng K, Wang J (2019). Transition from acute to chronic pain: evaluating risk for chronic postsurgical pain. Pain Physic.

[3] Savoia G, Ambrosio F, Paoletti F (2002). SIAARTI recommendations for the treatment of postoperative pain. Mine Anest.

[4] Bruce J, Quinlan J (2011). Chronic post surgical pain. Rev Pain.

[5] Fuzier R, Rousset J, Bataille B, Salces-y-Nédéo A, Maguès JP (2015). One half of patients reports persistent pain three months after orthopaedic surgery. Anaesth Crit Care Pain Med.

[6] Grosu I, de Kock M (2011). New concepts in acute pain management: strategies to prevent chronic postsurgical pain, opioid-induced hyperalgesia, and outcome measures. Anesthesiol Clin.

[7] Fletcher D, Stamer UM, Pogatzki-Zahn E (2015). Chronic postsurgical pain in Europe: an observational study. Eur J Anaesthesiol.

[8] Simanski CJ, Althaus A, Hoederath S (2014). Incidence of chronic postsurgical pain (CPSP) after general surgery. Pain Med.

[9] Smith WC, Bourne D, Squair J, Dean O, Chambers AW (1999). A retrospective cohort study of post mastectomy pain syndrome. Pain.

[10] Theunissen M, Peters ML, Bruce J, Gramke HF, Marcus MA (2012). Preoperative anxiety and catastrophizing: a systematic review and meta-analysis of the association with chronic postsurgical pain. Clin J Pain.

[11] Correll D (2017). Chronic postoperative pain: recent findings in understanding and management. F1000Res.

[12] Caumo W, Schmidt AP, Schneider CN (2002). Preoperative predictors of moderate to intense acute postoperative pain in patients undergoing abdominal surgery. Acta Anaesthesiol Scand.

[13] Wylde V, Dennis J, Beswick AD (2017). Systematic review of management of chronic pain after surgery. Br J Surg.

[14] Varrassi G, Hanna M, Macheras G (2017). Multimodal analgesia in moderate-to-severe pain: a role for a new fixed combination of dexketoprofen and tramadol. Curr Med Res Opin.

[15] Cho CH, Song KS, Min BW, Lee KJ, Ha E, Lee YC, Lee YK (2011). Multimodal approach to postoperative pain control in patients undergoing rotator cuff repair. Knee Surg Sports Traumatol Arthrosc.

[16] Amr YM, Yousef AA (2010). Evaluation of efficacy of the perioperative administration of venlafaxine or gabapentin on acute and chronic postmastectomy pain. Clin J Pain.

[17] Wong K, Phelan R, Kalso E, Galvin I, Goldstein D, Raja S, Gilron I (2014). Antidepressant drugs for prevention of acute and chronic postsurgical pain: early evidence and recommended future directions. Anesthesiology.

[18] Martinez V, Pichard X, Fletcher D (2017). Perioperative pregabalin administration does not prevent chronic postoperative pain: systematic review with a meta-analysis of randomized trials. Pain.

[19] Chaparro LE, Smith SA, Moore RA, Wiffen PJ, Gilron I (2013). Pharmacotherapy for the prevention of chronic pain after surgery in adults. Cochrane Database Syst Rev.

[20] Gold MS, Dastmalchi S, Levine JD (1997). α2-adrenergic receptor subtypes in rat dorsal root and superior cervical ganglion neurons. Pain.

[21] De Kock M, Lavand'homme P, Waterloos H (2005). The short-lasting analgesia and long-term antihyperalgesic effect of intrathecal clonidine in patients undergoing colonic surgery. Anesth Analg.

[22] Grigoras A, Lee P, Sattar F, Shorten G (2012). Perioperative intravenous lidocaine decreases the incidence of persistent pain after breast surgery. Clin J Pain.

[23] Andreae MH, Andreae DA (2012). Local anaesthetics and regional anaesthesia for preventing chronic pain after surgery. Cochrane Database Syst Rev.

[24] Kraychete DC, Sakata RK, Lannes Lde O, Bandeira ID, Sadatsune EJ (2016). Postoperative persistent chronic pain: what do we know about prevention, risk factors, and treatment. Braz J Anesthesiol.

[25] Richebé P, Capdevila X, Rivat C (2018). Persistent postsurgical pain: pathophysiology and preventative pharmacologic considerations. Anesthesiology.

[26] Pogatzki-Zahn EM, Segelcke D, Schug SA (2017). Postoperative pain-from mechanisms to treatment. PAIN Rep.

[27] Varrassi G, Yeam CT, Rekatsina M, Pergolizzi JV, Zis P, Paladini A (2020). The expanding role of the COX inhibitor/opioid receptor agonist combination in the management of pain. Drugs.

[28] Varrassi G, Fusco M, Skaper SD (2018). A pharmacological rationale to reduce the incidence of opioid induced tolerance and hyperalgesia: a review. Pain Ther.

[29] VanDenKerkhof EG, Hopman WM, Goldstein DH (2012). Impact of perioperative pain intensity, pain qualities, and opioid use on chronic pain after surgery: a prospective cohort study. Reg Anesth Pain Med.

[30] Varrassi G, Marinangeli F, Piroli A, Coaccioli S, Paladini A (2010). Strong analgesics: working towards an optimal balance between efficacy and side effects. Eur J Pain.

[31] O'Brien J, Pergolizzi J, van de Laar M (2013). Fixed-dose combinations at the front line of multimodal pain management: perspective of the nurse-prescriber. Nurs Res Rev.

[32] Gritsenko K, Khelemsky Y, Kaye AD, Vadivelu N, Urman RD (2014). Multimodal therapy in perioperative analgesia. Best Pract Res Clin Anaesthe.

[33] Clarke H, Poon M, Weinrib A, Katznelson R, Wentlandt K, Katz J (2015). Preventive analgesia and novel strategies for the prevention of chronic post-surgical pain. Drugs.

[34] Jian W, Rejaei D, Shihab A, Alston TA, Wang J (2018). The role of multimodal analgesia in preventing the development of chronic postsurgical pain and reducing postoperative opioid use. J Opioid Manag.

[35] Hanna M, Moon JY (2019). A review of dexketoprofen trometamol in acute pain. Curr Med Res Opin.

[36] Barbanoj MJ, Antonijoan RM, Gich I (2001). Clinical pharmacokinetics of dexketoprofen. Clin Pharmacokinet.

[37] Varrassi G, Coaccioli S, De-Andrés J (2019). Expert consensus on clinical use of an orally administered dexketoprofen plus tramadol fixed-dose combination in moderate-to-severe acute pain: a Delphi study. Adv Ther.

[38] Moore RA, Gay-Escoda C, Figueiredo R (2015). Dexketoprofen/tramadol: randomised double-blind trial and confirmation of empirical theory of combination analgesics in acute pain. J Headache Pain.

[39] Gay-Escoda C, Hanna M, Montero A (2019). Tramadol/dexketoprofen (TRAM/DKP) compared with tramadol/paracetamol in moderate to severe acute pain: results of a randomised, double-blind, placebo and active-controlled, parallel group trial in the impacted third molar extraction pain model (DAVID study). BMJ Open.

[40] Moore RA, McQuay HJ, Tomaszewski J (2016). Dexketoprofen/tramadol 25 mg/75 mg: randomised double-blind trial in moderate-to-severe acute pain after abdominal hysterectomy. BMC Anesthesiol.

[41] McQuay HJ, Moore RA, Berta A (2016). Randomized clinical trial of dexketoprofen/tramadol 25 mg/75 mg in moderate-to-severe pain after total hip arthroplasty. Br J Anaesth.

[42] Montero Matamala A, Bertolotti M, Contini MP (2017). Tramadol hydrochloride 75 mg/dexketoprofen 25 mg oral fixed-dose combination in moderate-to-severe acute pain: sustained analgesic effect over a 56-h period in the postoperative setting. Dru Tod (Barc).

[43] Derry S, Cooper TE, Phillips T (2016). Single fixed-dose oral dexketoprofen plus tramadol for acute postoperative pain in adults. Cochrane Database Syst Rev.

[44] Varrassi G, Alon E, Bagnasco M (2019). Towards an effective and safe treatment of inflammatory pain: a Delphi-guided expert consensus. Adv Ther.

